# Prueba nasofaríngea con resultado positivo tardío para COVID-19, un estudio de caso con correlación clínica y patológica

**DOI:** 10.1159/000521742

**Published:** 2022-01-26

**Authors:** Lei Zhang, Syam P. Vunnamadala, Shigeo Yagi, Riffat Meraj, Michele Carbone

**Affiliations:** ^a^Asociados en Patología de Anaheim, Anaheim, California, Estados Unidos; ^b^Centro Médico Regional de Anaheim, Anaheim, California, Estados Unidos; ^c^Hospital Médico de Riverside (Hospital Comunitario Parkview), Riverside, California, Estados Unidos; ^d^Departamento de Salud Pública de California, Laboratorio de Enfermedades Virales y Rickettsiales, Richmond, California, Estados Unidos; ^e^Oncología Torácica, Programa de Estudios de Biología del Cáncer, Centro del Cáncer de la Universidad de Hawái, Honolulu, Hawaii, Estados Unidos

## Abstract

**Antecedentes:**

Hay varias razones por las que las pruebas de PCR nasofaríngeas para la enfermedad por coronavirus 2019 (COVID-19) dan un resultado positivo tardío, no sólo en casos asintomáticos sino también en pacientes gravemente enfermos. Los atributos fisiopatológicos aún se desconocen. Exploramos esta posibilidad mediante el reporte de un caso.

**Presentación del caso:**

Un varón de 64 años con antecedentes de infección fúngica pulmonar, asma y enfermedad pulmonar obstructiva crónica (EPOC), diabetes y arteriopatía coronaria se presentó con dificultad respiratoria, fiebre e imágenes torácicas de opacidad esmerilada y engrosamiento intersticial reticular, altamente sospechoso de COVID-19. Sin embargo, las pruebas de hisopado nasofaríngeo fueron discordantemente negativas durante cuatro ocasiones en dos semanas, y los anticuerpos IgG para COVID-19 también fueron negativos. Sin embargo, el nivel de IgE en suero era elevado. No se identificaron otros patógenos. Sus síntomas se deterioraron a pesar del tratamiento con corticosteroides, antibióticos y broncodilatadores. Se realizaron un lavado broncoalveolar (LBA) y una biopsia en cuña a pulmón abierto para el diagnóstico de la etiología. Se demostró la presencia de neumonía organizativa fibrosante positiva para RNA viral COVID-19, y daños en las vías respiratorias caracterizados por un efecto citopático sospechoso de origen viral, inflamación mixta neutrofílica, linfoplasmática, histiocítica y eosinofílica, y fibrosis, además de los cambios esperados en el asma y la EPOC. Una semana después, las pruebas nasofaríngeas de COVID-19 repetidas en los días 40 y 49 resultaron positivas.

**Conclusión:**

Nuestro caso y la revisión de la literatura indican que el asma alérgica y el alto nivel de IgE asociado a ella, junto con la inhalación de corticosteroides, podrían contribuir a la positividad retardada del hisopado nasofaríngeo en las vías respiratorias superiores; la obstrucción crónica de las vías respiratorias relacionada con la EPOC y la adición de fibrosis provocaron dependencia del ventilador y un mal pronóstico en la neumonía por COVID-19, y deberían ser objeto de tratamiento junto con la terapia antiviral.

## Antecedentes

Desde que se aisló el coronavirus causante del síndrome respiratorio agudo severo (severe acute respiratory syndrome corona­virus 2, SARS-CoV-2) en Wuhan, China, a finales de 2019, la enfermedad por coronavirus 2019 (coronavirus disease 2019, COVID-19) se convirtió en una pandemia mundial [1]. En Estados Unidos se han confirmado más de 38 millones de casos de COVID-19, lo que representa entre 15 y 20% del recuento mundial. La reacción en cadena de la polimerasa con transcriptasa inversa (real-time polymerase chain reaction, RT-PCR) es la prueba de laboratorio más utilizada para detectar el SARS-CoV-2. Cuando se realiza en muestras de hisopado de las vías respiratorias superiores, se ha reportado una sensibilidad de 60–70% [1, 2]. Una proporción considerable de pacientes con COVID-19 puede mostrar un resultado inicial negativo en la RT-PCR. Además de las características de desempeño de los ensayos, el tiempo de muestreo y la fuente de la muestra contribuyen significativamente a la obtención de resultados «falsos» negativos [1, 3]. Presentamos un caso para discutir una posible explicación fisiopatológica, que no se ha reportado antes.

## Presentación del caso

### Hallazgos clínicos

Un hombre asiático de 64 años fue ingresado con fiebre (38.7 °C), tos que empeoraba y dificultad respiratoria durante 10 días. No es fumador y tiene una historia antigua de infección pulmonar por hongos hace más de 20 años, cuando era agricultor. Durante los últimos 3–4 años ha recibido atención en nuestro hospital por asma persistente y exacerbada, enfermedad pulmonar obstructiva crónica (EPOC), diabetes, enfermedad coronaria y cardiopatía congestiva. Su medicación a domicilio para el asma y la EPOC consiste en sulfato de albuterol HFA, 1.25 mg/3 ml cada 4 h según se requiera, Duoneb (una combinación de albuterol y bromuro de ipratropio) y Budesónida (Pulmicort, un medicamento similar a la cortisona, NEB 0.5 mg/2 ml) en inhalación. Durante una crisis asmática anterior, hace 13 meses, se administraron 40 mg de metilprednisona por vía intravenosa, cada 8 h, además de amoxicilina/ácido clavulánico. Durante la exacerbación de la EPOC hace dos años se administró prednisona, 40 mg por vía oral, diariamente. En el ataque actual se inició con dexametasona, 10 mg al día, Duoneb y tratamiento empírico con azitromicina en la consulta externa antes del ingreso. La prueba RT-PCR nasofaríngea para COVID-19 realizada en el consultorio médico fuera del hospital al quinto día del inicio de los síntomas fue negativa.

Al ingreso, su IMC era 21.10 kg/m^2^. La gasometría arterial reveló una presión parcial de oxígeno de 74 mmHg (normal 75–100), con una saturación de 96.1% (normal 92–98.5%), y una presión parcial de dióxido de carbono (pCO_2_) de 32 mmHg (normal 35–45); el pH era 7.48.

Las pruebas de laboratorio mostraron un recuento elevado de glóbulos blancos, hasta 22 × 10^3^/μl, con predominio de neutrófilos (neutrófilos 79% y 17 × 10^3^/μl, con 0–3% de eosinófilos), anemia moderada (8–10 g/dl) y trombocitosis reactiva (500–650 × 10^3^/L). La inmunoglobulina E estaba elevada (875, normal ≤114). El dímero D estaba ligeramente elevado (2.73 μg/ml, referencia <0.5), así como la prohormona N-terminal del péptido natriurético cerebral (NT-proBNP, 1270 pg/ml, referencia 0–900). El ácido láctico estaba elevado, 4.6 nmol/L (0.7–1.9). La proteína C reactiva también estaba elevada. La ferritina y la procalcitonina estaban dentro del límite normal. Todas las pruebas de antígenos, anticuerpos y cultivos para patógenos comunes de enfermedades infecciosas como la tuberculosis, la hepatitis, el VIH, bacterias (incluyendo *Streptococcus*, *Legionella*, etc.), hongos (incluyendo coccidioidomicosis, *Cryptococcus*, etc.) fueron negativos. Tampoco había autoanticuerpos (anticuerpos antinucleares, anticuerpos citoplasmáticos antineutrófilos). Las pruebas nasofaríngeas repetidas en los días 10, 13 y 14 del inicio de los síntomas, realizadas en el hospital tras el resultado negativo inicial del día 5, fueron todas negativas, y el anticuerpo IgG para COVID-19 del día 16 también fue negativo.

**Fig. 1:**
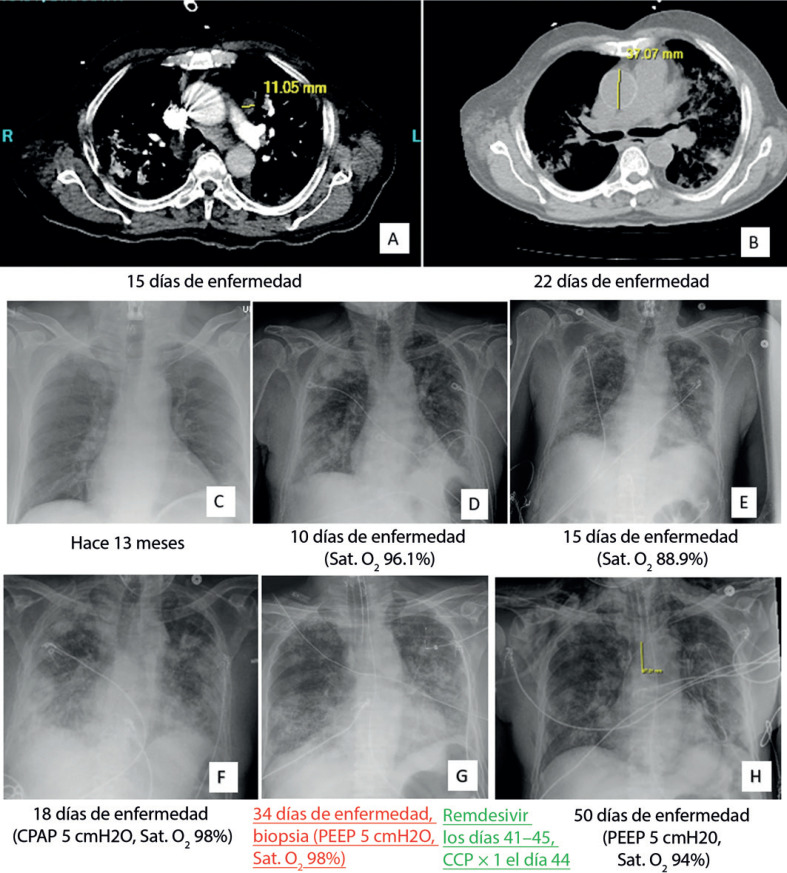
Imágenes de TAC y radiografía torácica. El panel superior (**a** y **b**) muestra la TAC, y los paneles medio e inferior (**c–h**) muestran la radiografía torácica. Las imágenes de TAC de los días 15 (**a**) y 22 (**b**) muestran infiltrados pulmonares bilaterales irregulares con predominio de componentes de vidrio esmerilado, sospechosos de neumonía, incluso COVID-19. La enfermedad sobreañadida se localiza mayoritariamente en la periferia (**d–h**), en contraste con la crisis asmática previa de hace 13 meses (**c**) (panel medio e inferior, radiografía de tórax). La enfermedad se estabilizó inicialmente con corticosteroides, antibióticos (incluyendo azitromicina) y tratamiento broncodilatador; el paciente no fue intubado hasta el día 15 (**d** y **e**). La condición se deterioró el día 18 (**f**) y la hipoxemia requirió ventilación y un procedimiento diagnóstico de lavado broncoalveolar y biopsia pulmonar en cuña (**g**). Tras la confirmación de COVID-19, el remdesivir, plasma de convaleciente de COVID-19 y el corticosteroide continuo, así como el broncodilatador, condujeron a una ligera disolución de la opacidad pulmonar (**h**).

La TAC y la radiografía torácica mostraron opacidades con aspecto de vidrio molido con base periférica y consolidación, en contraste con las sombras de localización central durante la crisis asmática hace 13 meses (Fig. 1). Éste es un rasgo de imagen establecido de infección activa por COVID-19, pero sigue siendo inespecífico, porque otras infecciones y enfermedades del espacio aéreo tienen una apariencia similar. No se detectaron embolias pulmonares en la TAC.

Se le aisló como precaución debido a la incertidumbre por el COVID-19. Se trató con antibióticos empíricos, corticoides inhalados y broncodilatadores, así como esteroides sistémicos, además de aspirina, diuresis y medicamentos para la diabetes. Los principales medicamentos para tratar la enfermedad pulmonar son:

Antibióticos: azitromicina (500 mg diarios por vía oral, días 5 a 14), ceftriaxona (1 g, iv, días 9 a 15), Zosyn (3.375 g, días 15 a 19), cefepime (1 g, días 21 a 31), fluconazol (200 mg, días 22 a 33), meropenem (1 g, días 36 a 40) y vancomicina (750 a 1000 mg, días 36 a 40).
Medicamentos inhalados con corticosteroides y broncodilatadores para el asma y la EPOC: Advair (Flutica-Salmet, una combinación de esteroides y broncodilatadores, dosificador 250–50 μg/inhalación, días 11–25), Budesónida (Pulmicort, medicamento similar a la cortisona, 0.5 mg, 2 por día en inhalación, días 16–50), Duoneb (Ipratr-albut, un broncodilatador que contiene medicamentos betaadrenérgicos y anticolinérgicos, 0.5–3 mg/3 ml, cada 4 h, días 5–50).
Esteroides sistémicos: dexametasona (10 mg los días 5–10, 6 mg los días 11–37), succinato de metilprednisolona (Solu-Medrol, 40 mg, días 16–20, 24–33 y 37–50), y Prednisona (10 mg, días 33–37).

La enfermedad se estabilizó inicialmente (Fig. 1e). En el día 18, los síntomas se deterioraron con un descenso de la saturación de O_2_ de 74 a 54 mmHg. La radiografía torácica mostró un aumento en la opacidad pulmonar bilateral (Fig. 1f). Los medicamentos indicados anteriormente, incluyendo antifúngicos, antibacterianos, broncodilatadores, esteroides inhalados y sistémicos, dejaron de ser útiles para mejorar los síntomas (Fig. 1g).

En ese momento se hizo un lavado broncoalveolar (LBA) para identificar una etiología infecciosa y se requirió una biopsia a pulmón abierto en cuña para descartar una enfermedad pulmonar intersticial.

### Hallazgos patológicos

**Fig. 2:**
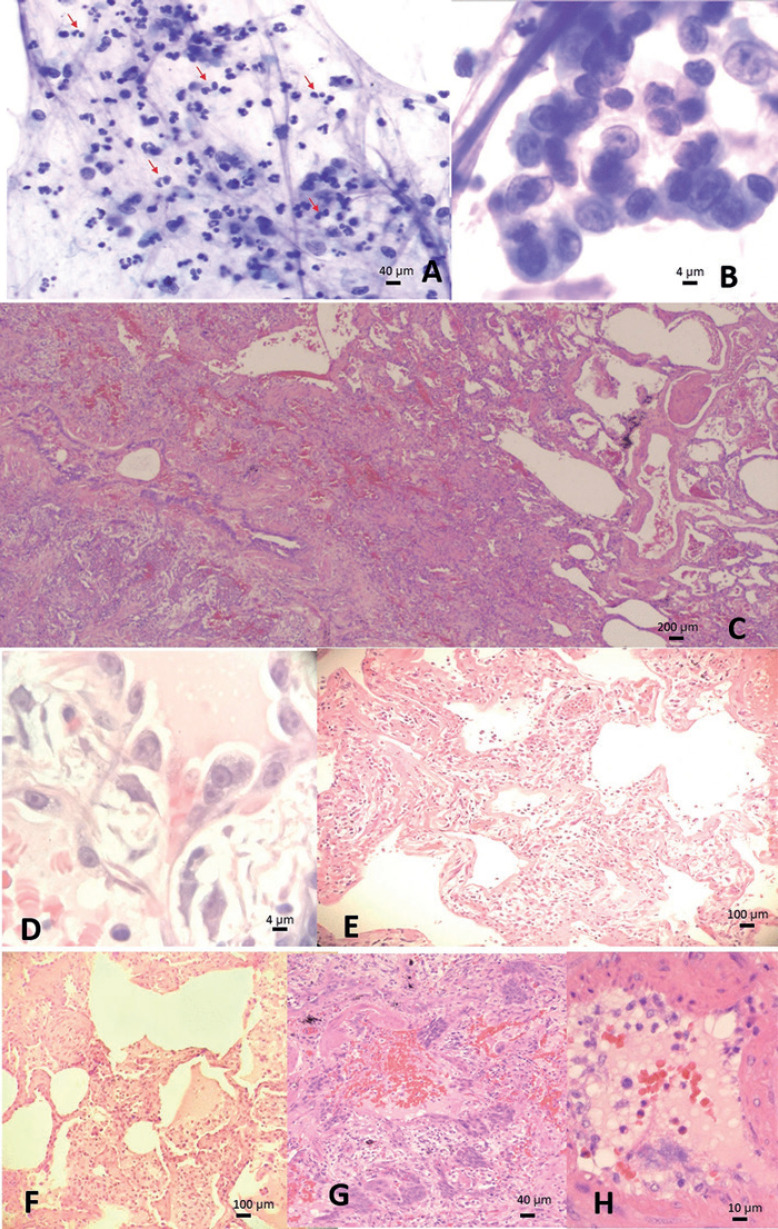
Hallazgos patológicos del lavado broncoalveolar y de la biopsia pulmonar en cuña. Lavado broncoalveolar que muestra (**a**) inflamación con predominio neutrofílico combinado con algunos eosinófilos (flecha roja, compatible con historia previa de asma), y (**b**) neumocitos reactivos con efecto citopático sospechoso de origen viral; (**c–h**) biopsia pulmonar en cuña que revela cambios asmáticos, con pared bronquiolar engrosada (**c**, izquierda) y tapón de moco (**c**, arriba a la derecha), y EPOC que se caracteriza por enfisema con alveolos dilatados distales al bronquiolo, sin fibrosis (**c**, derecha) y metaplasia escamosa reparadora del daño crónico (**c**, abajo a la izquierda), así como neumonía activa de reciente aparición con fibrosis alveolar e intersticial (**c**, medio), neumocitos tipo II con posible efecto citopático viral (**d**), neumonía aguda con daño alveolar (e), exudado edematoso alveolar, fibrosis intraalveolar (**f**), escasas células sincitiales en medio de metaplasia escamosa (**g**) y lesión endotelial (**h**). Las imágenes se tomaron con un microscopio Olympus modelo BX45TF, cámara Olympus modelo DP71 y software Olympus CellSens a una resolución de 72 ppp, y se procesaron en Adobe Photoshop CS5.1 con una resolución de 300 ppp.

El LBA mostró varios neumocitos con disposición acinar que mostraban núcleos agrandados, nucleolos prominentes, cromatina abierta y acentuación leve de la cromatina a la membrana nuclear (Fig. 2b) en un fondo de inflamación histiocítica neutrofílica y ocasionales eosinófilos (Fig. 2a).

La biopsia en cuña de la língula pulmonar izquierda (Fig. 2c) reveló un bronquiolo central con pared de músculo liso engrosada y un tapón de moco, hallazgos consistentes con la historia conocida de asma. La presencia de metaplasia escamosa central y enfisema periférico indica daño crónico a las vías respiratorias y obstrucción que sugiere EPOC por episodios repetidos de asma. Además, hay una lesión activa centrada en la vía respiratoria que causa 40–50% de colapso alveolar, caracterizada por neumonía organizativa fibrosante con daño alveolar (Fig. 2e), exudados edematosos intraalveolares, fibrosis intraalveolar que se extiende a los intersticios (Fig. 2f), asociada con escasas células sincitiales en focos de metaplasia escamosa (Fig. 2g) y lesión endotelial capilar (Fig. 2h) acompañada de inflamación mixta con neutrófilos, células linfoplasmáticas, histiocitos y eosinófilos en todo el proceso. De nuevo, de forma similar a la citología, se observan grandes neumocitos reactivos que recubren los alveolos hemorrágicos, mostrando nucleolos prominentes, cromatina abierta y acentuación de la membrana nuclear, sospechosos de efecto citopático viral (Fig. 2d). La infección por citomegalovirus, adenovirus y virus del herpes se excluyó por tinciones inmunohistoquímicas.

Se detectó SARS-CoV-2 tanto en el LBA (Quest Laboratory) como en la biopsia pulmonar en cuña (Laboratorio de Enfermedades Virales y Rickettsiales (VRDL), Departamento de Salud Pública, California) por RT-PCR. No se detectó el virus sincitial respiratorio (VRS), influenza A o B, virus de la parainfluenza o adenovirus por PCR (Quest Laboratory). Los tres laboratorios (la RT-PCR del hisopado nasofaríngeo realizada por Fulgent Genetics, la muestra de LBA analizada por Quest Laboratory, la presencia de COVID-19 en el tejido de la biopsia pulmonar fijada en parafina y verificada por VRDL) utilizan los mismos conjuntos de *primers* exclusivos para el SARS-CoV-2 diseñados por el Centro para el Control y la Prevención de Enfermedades (Center for disease control and prevention, CDC) de los EE. UU. Se confirma la neumonía por COVID-19. Pruebas repetidas de RT-PCR nasofaríngea fueron positivas el día 40 y el día 49 de la enfermedad.

### Seguimiento

Tras el diagnóstico de neumonía por COVID19, el paciente recibió Remdesivir (200 mg, día 41–45) y plasma de convaleciente de COVID-19 (una unidad, el día 44), además de continuar los corticosteroides, broncodilatadores y el tratamiento de apoyo. La granulocitosis disminuyó hasta un punto mínimo de 12 K/μl tras el tratamiento. La opacidad pulmonar comenzó a disolverse lentamente (Fig. 1h) pero persistió la hipoxemia, que aún requirió ventilación. Se aislaron bacterias gramnegativas del catéter central el día 50. La vía central infectada se sustituyó inmediatamente y el paciente fue tratado con meropenem, se le trasladó a otro hospital para continuar su cuidado. Lamentablemente, el paciente sucumbió a las complicaciones sépticas dos meses después de la aparición de la enfermedad.

## Discusión y conclusión

### Papel del asma alérgica, la EPOC y otras condiciones en la susceptibilidad al COVID-19 y la gravedad de la infección

Existen evidencias clínicas contradictorias sobre si el asma es un factor de susceptibilidad para la infección por COVID-19. Sin embargo, tras separar el asma alérgica de la no-alérgica, no se observa una asociación estadísticamente significativa con los síntomas graves de COVID-19 en los pacientes con asma alérgica [4, 5].

La infección por COVID-19 se inicia principalmente en la nariz; luego, el virus llega a la orofaringe y es aspirado a la parte inferior del pulmón. Por esta razón, la nasofaringe es un lugar común para el análisis de COVID-19. La enzima convertidora de angiotensina 2 (angiotensin-converting enzyme, ACE2) y la serina proteasa transmembranal 2 (transmembrane serine protease 2, TMPRSS2) son los principales receptores de entrada para el SARS-CoV-2 a las células humanas. Un estudio reveló una menor expresión de ACE2 en muestras de hisopado nasal de personas con asma alérgica, junto con una disminución progresiva de la expresión de ACE2 relacionada con una mayor sensibilización a la IgE [6]. Además, se ha planteado la hipótesis de que las citocinas de respuesta inflamatoria de tipo II (IL-4, −5 y −13) y la acumulación de eosinófilos que se observa en el asma pueden ser protectoras contra el COVID-19 [4].

A diferencia del asma, la EPOC es un factor de riesgo establecido para el COVID-19, y está asociada con la gravedad de la infección. Esto se relaciona posiblemente con una expresión elevada de ACE2 y una defensa subóptima del huésped, así como con el daño vascular [7]. Entre los pacientes con asma, el nivel de expresión de ACE2 y TMPRSS2 en las células de esputo del tracto respiratorio inferior fue mayor en pacientes de sexo masculino, raza afroamericana y antecedentes de diabetes mellitus [8].

La inhalación de corticosteroides, tratamiento tanto para el asma como para la EPOC, se asoció con una menor expresión de ACE2 y TMPRSS2 [4, 9]. Se ha demostrado que la inhalación de corticosteroides suprime la replicación del coronavirus, así como la producción de citoquinas, y disminuye la expresión de los genes *ACE2* y *TMPRSS2* en pacientes con asma [4].

### Un resultado positivo tardío de la prueba COVID-19 podría relacionarse con el asma, niveles elevados de IgE e inhalación de corticosteroides

En nuestro caso, cuatro pruebas nasofaríngeas practicadas en dos semanas desde el inicio de los síntomas fueron negativas para COVID-19. La probabilidad de una tasa de falsa omisión para cuatro pruebas nasofaríngeas negativas en un contexto de prevalencia del COVID-19 de 15–20% es de 0.01–0.03, según un modelo bayesiano [10]. Sin embargo, los resultados nasofaríngeos negativos fueron discordantes con los hallazgos de imagen del pulmón.

La carga viral parece seguir una distribución normal en la mayoría de los casos recuperados de COVID-19, y la ventana diagnóstica de la prueba PCR se encuentra entre −2 y 18 días del inicio de los síntomas. El virus no suele ser detectable después de 20 días de enfermedad mediante la prueba nasofaríngea [3]. La prueba serológica empieza a ser positiva a los 7 días [3]. En nuestro caso, las pruebas de PCR nasofaríngeas fueron negativas en los días 5, 10, 13 y 14. Sin embargo, fueron positivas el día 40 y 49 tras el inicio de los síntomas. El anticuerpo IgG anti-COVID-19 fue negativo el día 16. Todo ello indica una larga latencia de la infección por el virus en el tracto respiratorio superior de este paciente.

Los antecedentes de asma del paciente, los niveles elevados de IgE y la inhalación de corticosteroides podrían asociarse con niveles menores del receptor ACE2 e impedir la detección inicial del virus en la zona nasofaríngea [4, 6]. Por otra parte, la expresión de los receptores del SARS-CoV-2 se eleva en casos de EPOC y de diabetes, así como en pacientes de sexo masculino [7, 8]. Dicha sobreexpresión podría ser más prominente en la parte inferior del pulmón, donde la EPOC se manifiesta patológicamente. Esto puede reflejar una carga de virus por debajo del rango detectable en el tracto respiratorio superior, mientras que el virus permanece «oculto» en el pulmón inferior y se manifiesta en la opacidad periférica en las imágenes. El virus SARS-CoV-2 se identificó finalmente por primera vez mediante un lavado broncoalveolar, que tiene una sensibilidad elevada para el COVID-19, de 90%, en comparación con el 70% de las muestras nasofaríngeas y de esputo [10].

En la pandemia, la capacidad de los hospitales está desbordada y los recursos para realizar las pruebas son limitados, especialmente al principio de la enfermedad [1]. El reconocimiento de la susceptibilidad diferencial en asociación con enfermedades preexistentes y la aplicación de una estrategia de detección evitaría descartar erróneamente el COVID-19 con base en pruebas de diagnóstico falsamente negativas. El LBA tiene la mayor sensibilidad reportada, pero puede aerosolizar partículas infecciosas. No se ha reportado la sensibilidad del esputo obtenido de pacientes intubados por succión traqueal profunda a través de un circuito cerrado de ventilación, pero puede representar una alternativa práctica al LBA [10].

### Patología, imagen y correlación clínica

Se han reportado efectos citopáticos virales como nucleolos prominentes, núcleos abiertos con marginación periférica de la cromatina y escasas células sincitiales en la infección por COVID-19. Aunque estas características citológicas son altamente sugestivas de COVID-19, son inespecíficas. Lo mismo ocurre con las imágenes radiográficas. Las opacidades consolidadas periféricas y las opacidades difusas en vidrio esmerilado son signos de imagen establecidos de la infección activa por COVID-19, pero otras infecciones/enfermedades del espacio aéreo podrían tener un aspecto similar. El diagnóstico definitivo aún depende de la identificación de la secuencia de RNA del SARS-CoV-2.

El inicio de la neumonía por COVID-19 en este paciente se asocia con granulocitosis en la sangre periférica y la presencia de trampas extracelulares de neutrófilos en el pulmón en ausencia de otra etiología infecciosa. No observamos hemofagocitosis en nuestra muestra. La granulocitosis podría deberse a un desplazamiento de los neutrófilos del reservorio marginado al circulante tras el tratamiento con corticosteroides. Sin embargo, la leucocitosis remitió parcialmente tras el tratamiento antiviral con Remdesivir y plasma de convaleciente, mientras el paciente seguía bajo tratamiento continuo con corticosteroides. Esto sugiere que la granulocitosis podría ser una reacción del huésped a la infección activa por el SARS-CoV-2 en un subconjunto de pacientes, probablemente con comorbilidad de EPOC. Un reporte ha relacionado las trampas aberrantes de neutrófilos en el espacio extracelular, las llamadas «NET», con la presencia de daños orgánicos en el parénquima alveolar y las vías respiratorias [11]. La granulocitosis periférica también se asocia a un mal pronóstico en los casos de COVID-19 [11]. El papel de las «NET» en el COVID-19 requiere más estudios.

La biopsia pulmonar en cuña en el día 34 del inicio de la enfermedad mostró un triple hallazgo de lesión celular respiratoria, daño vascular y fibrosis, similar a la patología pulmonar de COVID-19 reportada previamente [12]. Sin embargo, la biopsia pulmonar en cuña de nuestro caso mostró una abundante infiltración neutrofílica, que sólo se ha reportado en un par de casos de autopsia [11]. Esto contrasta con el patrón de infiltración de células linfoplasmáticas y macrófagos ampliamente descrito y asociado con la neumonía por COVID-19 [12]. Cabe destacar que en la revisión sistemática, que analizó 198 casos por patología [12], sólo cuatro pacientes tenían EPOC y un paciente tenía asma; ninguno de ellos tenía una combinación de asma y EPOC, y tampoco se conocía el nivel de IgE en la sangre.

En la biopsia pulmonar en cuña, las células endoteliales se desprendieron de la pared vascular y los pequeños vasos presentaban fugas que provocaron hemorragias en los alveolos. El daño endotelial podría explicar la ligera elevación del dímero D. No se identificó inflamación perivascular significativa ni vasculitis. No se observó la presencia significativa de membrana hialina en los alveolos, un signo de daño alveolar en fase inicial. Más bien, hubo reparación del daño alveolar, incluyendo metaplasia escamosa (relacionada tanto con la infección actual como con la larga historia de EPOC) y fibrosis en evolución, que suelen producirse después de 28 días de enfermedad [12]. Los hallazgos patológicos de neumonía organizativa fibrosante atestiguan la cronología de la infección, indicando que la replicación activa del SARS-CoV-2 en el pulmón periférico se produjo antes de las cuatro pruebas nasofaríngeas negativas. Estos cambios reparadores fibrosantes aumentaron el volumen del pulmón no aireado, lo que obligó al paciente a depender del ventilador. Un tratamiento que ayude a disolver la fibrosis podría favorecer una supervivencia más prolongada [1].

## Conclusión

El reconocimiento de la susceptibilidad diferencial en asociación con algunas enfermedades preexistentes puede ayudar a evitar que se descarte erróneamente el COVID-19 con base en pruebas diagnósticas falsamente negativas. Nuestro caso y la revisión de la literatura indican que: (1) El asma alérgica y los altos niveles de IgE asociados, junto con la inhalación de corticosteroides, podrían contribuir a la positividad tardía del hisopado nasofaríngeo en la vía respiratoria superior. (2) La obstrucción crónica de las vías respiratorias relacionada con la EPOC y la adición de fibrosis indujeron dependencia del ventilador y mal pronóstico en la neumonía por COVID19, y deberían recibir tratamiento terapéutico junto con la terapia antiviral.

## Disponibilidad de datos y materiales

Todos los datos generados o analizados durante este estudio se incluyen en este artículo. Todos los datos y materiales están disponibles para compartirse si es necesario. Por favor, póngase en contacto con el autor corresponsal.

## Agradecimientos

Agradecemos a los familiares del paciente, quienes compartieron la información clínica con la esperanza de salvar vidas. Agradecemos sinceramente a los revisores y editores, cuyas sugerencias críticas mejoran nuestro reporte.

## Financiamiento

M.C. reporta financiamiento del Instituto Nacional de Ciencias de la Salud Ambiental (NIEHS) 1R01ES030948–01, el Instituto Nacional del Cáncer (NCI) 1R01CA237235–01A1 y 1R01CA198138, y de la Fundación UH a través de donaciones de: Riviera United-4-a Cure, la dotación de la familia Melohn, Honeywell International Inc., la Fundación Germaine Hope Brennan, y la Fundación de la Familia de Maurice and Joanna Sullivan. M.C. es un patólogo certificado, que ofrece consultas de patología pleural, incluyendo servicio médico legal.

## Consentimiento para la publicación

Se obtuvo el consentimiento informado por escrito de los familiares del paciente para publicar esta información.

## Conflictos de interés

Los autores declaran que no tienen conflictos de interés.

## Información sobre licencias

Lei Zhang, Syam P. Vunnamadala, Shigeo Yagi, Riffat Meraj, Michele Carbone: Delayed positive COVID19 nasopharyngeal test, a case study with clinical and pathological correlation. BMC Pulm Med. 2021;21(1):278 (DOI: 10.1186/s12890–021–01643-y). ^©^ 2021 Los Autores (traducción; abreviaturas, contribuciones de los autores, aprobación ética y consentimiento para participar, consentimiento para publicación, nota del editor abreviadas), protegido por CC BY 4.0 (https://creativecommons.org/licenses/by/4.0/deed.es).

## References

[1] Carbone M, Lednicky J, Xiao SY, Venditti M, Bucci E (2021). Coronavirus 2019 infectious disease epidemic: where we are, what can be done and hope for. J Thorac Oncol.

[2] Ai T, Yang Z, Hou H, Zhan C, Chen C, Lv W (2020). Correlation of chest CT and RT-PCR testing for coronavirus disease 2019 (COVID-19) in China: a report of 1014 cases. Radiology.

[3] Younes N, Al-Sadeq DW, AL-Jighefee H, Younes S, Al-Jamal O, Daas HI (2020). Challenges in laboratory diagnosis of the novel coronavirus SARS-CoV-2. Viruses.

[4] Hughes-Visentin A, Paul ABM (2020). Asthma and COVID-19: what do we know now. Clin Med Insights Circ Respir Pulm Med.

[5] Robinson LB, Fu X, Bassett IV, Triant VA, Foulkes AS, Zhang Y (2020). COVID-19 severity in hospitalized patients with asthma: a matched cohort study. J Allergy Clin Immunol Pract.

[6] Jackson DJ, Busse WW, Bacharier LB (2020). Association of respiratory allergy, asthma, and expression of the SARS-CoV-2 receptor ACE2. J Allergy Clin Immunol.

[7] Higham A, Mathioudakis A, Vestbo J (2020). COVID-19 and COPD: a narrative review of the basic science and clinical outcomes. Eur Respir Rev.

[8] Peters MC, Sajuthi S, Deford P (2020). COVID-19-related genes in sputum cells in asthma: relationship to demographic features and corticosteroids. Am J Respir Crit Care Med.

[9] Janson C (2020). Treatment with inhaled corticosteroids in chronic obstructive pulmonary disease. J Thorac Dis.

[10] Raschke R.A., Curry S.C., Glenn T., Gutierrez F., Iyengar S. (2020). A Bayesian analysis of strategies to rule out coronavirus disease 2019 (COVID-19) using reverse transcriptase-polymerase chain reaction. Arch Pathol Lab Med.

[11] Barnes BJ, Adrover JM, Baxter-Stoltzfus A, Borczuk A, Cools-Lartigue J, Crawford JM (2020). Targeting potential drivers of COVID-19: neutrophil extracellular traps. J Exp Med.

[12] Polak SB, Van Gool IC, Cohen D, von der Thüsen JH, van Paassen J (2020). A systematic review of pathological findings in COVID-19: a pathophysiological timeline and possible mechanisms of disease progression. Mod Pathol.

